# Ultrasound-guided cranial multifidus cervicis plane block in dogs: a cadaveric study

**DOI:** 10.3389/fvets.2026.1835968

**Published:** 2026-05-26

**Authors:** Ariel Cañón Pérez, Jonatan Terminiello, Jaime Viscasillas Monteagudo, Natalia Aguilar Gallego, Rocío Fernández Parra, José Ignacio Redondo García, Cristina Bonastre Ráfales

**Affiliations:** 1 Department of Animal Pathology, Faculty of Veterinary Medicine, University of Zaragoza, Zaragoza, Spain; 2 Experimental Surgery Unit (ESU), Vall d'Hebron Institut de Recerca (VHIR), Vall d'Hebron, Hospital Universitari, Barcelona, Spain; 3 Cátedra de Anatomía Descriptiva y Topográfica. Facultad de Ciencias Veterinarias, Universidad Nacional de La Plata (UNLP), La Plata, Argentina; 4 Hospital Veterinario AniCura Valencia Sur, Silla, Spain; 5 Departamento de Medicina y Cirugía Animal, Facultad de Veterinaria y Ciencias Experimentales, Hospital Veterinario de Referencia UCV, Universidad Católica de Valencia San Vicente Mártir, Valencia, Spain; 6 Departamento Medicina y Cirugía Animal, Facultad de Veterinaria y Ciencias Experimentales, Universidad Católica de Valencia San Vicente Mártir, Valencia, Spain; 7 Departamento de Medicina y Cirugía Animal, Facultad de Veterinaria, Universidad Cardenal Herrera-CEU, CEU Universities, Valencia, Spain; 8 Instituto Universitario de Investigación Mixto Agroalimentario de Aragón (IA2), University of Zaragoza, Zaragoza, Spain

**Keywords:** canine, cervical dorsal rami, cervical spine, interfascial plane approach, multimodal analgesia, regional anesthesia, sonoanatomy

## Abstract

**Introduction:**

Management of pain in the canine cervical spine is a challenge where regional anesthesia of the cervical dorsal branches via an interfascial plane approach can improve multimodal analgesia. This study aimed to describe the ultrasound-guided multifidus cervicis plane (US-MCP) approach in dogs and evaluate its distribution and nerve staining.

**Methods:**

An anatomical and sonoanatomical study was followed by an experimental phase using 15 ultrasound-guided injections performed at the C3 level (0.3 mL kg^−1^ of a methylene blue, iodinated contrast, and saline mixture) in canine cadavers. Contrast spread and nerve staining were evaluated using computed tomography (CT) and subsequent anatomical dissection.

**Results:**

Sonoanatomical landmarks were consistently identified in all cases. CT contrast spread reached the C1–C2 levels cranially and C4–C5 caudally in most cases. Contrast distribution reached the dorsal midline in 12/15 injections. Epidural migration occurred in 4/15 cases, mainly at the C2–C3 level. Dissection confirmed accurate interfascial deposition in all specimens. Nerve staining was 100% effective (15/15) for the dorsal branches of C2 and C3 and 60% in the case of C4 (9/15). However, success rates decreased caudally and staining was observed in 13.3% (2/15) of C5 nerves, and was absent (0/15) at C6.

**Conclusion:**

The described US-guided MCP approach is a feasible and highly specific technique for the desensitization of the cranial dorsal cervical region. Consistent staining of the C2 and C3 dorsal branches, and potentially C4, supports its potential as a promising tool for integration into multimodal analgesia protocols. Clinical studies are required to evaluate its safety and analgesic efficacy.

## 1 Introduction

Managing cervical pain remains a frequent clinical challenge in both human medicine (1) and veterinary medicine (2). Such pain can be mechanical, neuropathic, or referred, and it is associated with different structures (the annulus fibrosus, the dorsal longitudinal ligament, spinal nerve roots, meninges, articular facets, and vertebral bones) (3). Intervertebral disc disease has an estimated lifetime prevalence of approximately 3.5% in the general population in dogs (4), and up to 25% of these cases affect the cervical spine (5). The therapeutic approach depends on the etiology; however, regardless of medical or surgical treatment, analgesic management must be multimodal (3).

Cervical nerves, like all spinal nerves, are divided into a dorsal branch, a ventral branch, and a communicating branch (ramus communicans). The dorsal branch is distributed to the epaxis, specifically, to all structures located dorsally and laterally to the vertebral arches (6). This distribution encompasses the musculature of this region and the respective cutaneous innervation, with suggestive evidence of nerve projections toward the vertebrae, the ligamentous complex, and the dura mater (7). Therefore, these branches constitute the primary target of locoregional blocks intended for analgesia of the laterodorsal cervical structures.

The continuous development of locoregional anesthesia techniques has provided anesthesiologists with new tools to optimize perioperative analgesia. The incorporation of ultrasonography has increased the safety of these procedures by reducing potential complications such as vascular puncture or inadvertent intravascular injection and it has also allowed for a reduction in the required anesthetic volumes (8). In recent years, interfascial plane blocks have gained relevance by providing analgesia across extensive regions through the injection of local anesthetics into interfascial planes (9–11).

In the context of spinal surgery in veterinary medicine, multiple locoregional techniques have been described. Among these, the erector spinae plane (ESP) block is the most extensively researched in dogs, in both cadaveric studies (12, 13) and clinical trials (14–18). However, literature regarding specific blocks for cervical surgery remains scarce.

In human medicine, in addition to the ESP block, other alternative interfascial plane blocks have been described for cervical surgery, such as the cervical retrolaminar block, the inter-semispinal plane block, and the multifidus cervicis plane (MCP) block (19). The MCP block consists of injecting the anesthetic into the fascial plane between the multifidus cervicis and semispinalis capitis muscles, and it has demonstrated efficacy in both cervical surgery (20) and the treatment of chronic cervical pain (21). A similar approach was recently described in veterinary medicine, specifically an ultrasound-guided (US) inter-transversospinalis plane block at the C5 level, in a cadaveric study (22) that showed promising results.

From a clinical perspective, the implementation of locoregional techniques in cervical surgery is less established. Mangas-Ballester et al. (23) recently employed a cervical plexus block [a technique previously described in canine cadavers (24, 25)] for ventral slots, and also presented a conference communication comparing its efficacy to continuous rate infusion (26). In contrast, to the authors' knowledge, evidence for dorsal approaches is limited to isolated case reports, such as the ESP block (27) or the articular process block presented at a conference (28). Regarding the MCP block, its clinical application was recently described in a case report (29). This limited evidence underscores the critical need to expand the scientific knowledge supporting both the development and implementation of new locoregional techniques in this anatomical region.

The objective of the present study was to describe an ultrasound-guided approach to the multifidus cervicis plane (US-MCP) in canine cadavers, using a cranial approach, to reach the dorsal branches of the cranial cervical spinal nerves (C2 to C4). The hypothesis was that this technique would allow for adequate distribution toward these branches.

## 2 Materials and methods

This prospective anatomical cadaveric study was approved by the Animal Experimentation Ethics Committee of the Catholic University of Valencia San Vicente Mártir (CEEAUCV2403).

The experimental design was divided into three consecutive phases. In Phase I, bibliographic information regarding dorsal cervical anatomy was contrasted through detailed dissection aimed at the direct recognition of the involved structures in two cadavers. In Phase II, the sonoanatomy of the dorsal cervical region was assessed, and the US-guided injection technique was standardized in one cadaver, which also included an evaluation of the injected volume distribution. Finally, in Phase III, the study of this distribution was completed following the performance of the standardized US-MCP block in eight additional cadavers.

The study initially assessed 12 adult canine cadavers, euthanized for reasons unrelated to this study. Exclusion criteria were established based on visual inspection for external cervical anomalies and the inability to achieve adequate ultrasound visualization of the target anatomical landmarks during a preliminary examination. One cadaver was excluded due to poor image quality (attributed to a combination of excessive adipose tissue and post-mortem changes) that precluded the ultrasound visualization of the muscles and interfascial planes of interest. Consequently, 11 cadavers were finally included in the study. The final sample presented a median weight of 23.0 kg (range: 12.0–37.5 kg) and a median body condition score of 3/5 (range: 2/5–4/5) according to a 5-point scale. Two fresh cadavers were used in Phase I and subsequently fixed for anatomical dissection. The remaining cadavers (n = 9) were kept frozen (at −20 °C) and thawed at room temperature for 48 h prior to use. Demographic data are presented in Table 1.

**Table 1:** Demographic data and phase distribution of the 11 canine cadavers included in the study.

Id	Phase	Breed	BCS (1–5 scale)	Sex	Weight (kg)
ANAT01	I	Pitbull terrier	3	**♂**	23.0
ANAT02	I	Poodle	3	♀	12.0
CAD01	II	German shepherd	3	♀	37.5
CAD02	III	Mixed breed	2	♀	13.3
CAD03	III	Mixed breed	3	♀	13.6
CAD04	III	German shepherd	3	**♂**	36.0
CAD05^*^	III	Mixed breed	3	♀	16.4
CAD06	III	Mixed breed	3	**♂**	26.0
CAD07	III	Mixed breed	4	♀	29.3
CAD09	III	Boxer	2	**♂**	16.0
CAD10	III	Labrador retriever	3	♀	36.0

^*^Unilateral injection.

### 2.1 Phase I: anatomical dissection and bibliographic correlation

Due to the limited detailed description available in the current literature regarding the specific topography of the dorsal branches of the cervical nerves (DBCNs), an exhaustive anatomical study was conducted with the dual objective of contrasting the findings with published evidence and refining the anatomical knowledge of the region. The analysis focused on the topography and distribution of the C2 to C5 DBCNs, which were dissected by an anatomist (J.T).

Cadaver preparation consisted of placing flexible cannula into both internal carotid arteries and external jugular veins. Subsequently, intra-arterial perfusion was performed using a fixing solution routinely used for anatomical preparations by the Department of Descriptive and Topographical Anatomy (UNLP, Argentina), composed of 7% formaldehyde, 2% neutral glycerin, 5% of a 1.8% sodium chloride solution, and water. Following fixation, red dye was injected for the arteries and blue dye for the veins. Layer-by-layer dissection by was performed at 48 h after fixation and vascular filling, with the process documented through photographic images. General surgery instrumentation was used for the dissection.

### 2.2 Phase II: sonoanatomy and technique standardization

**Figure 1:**
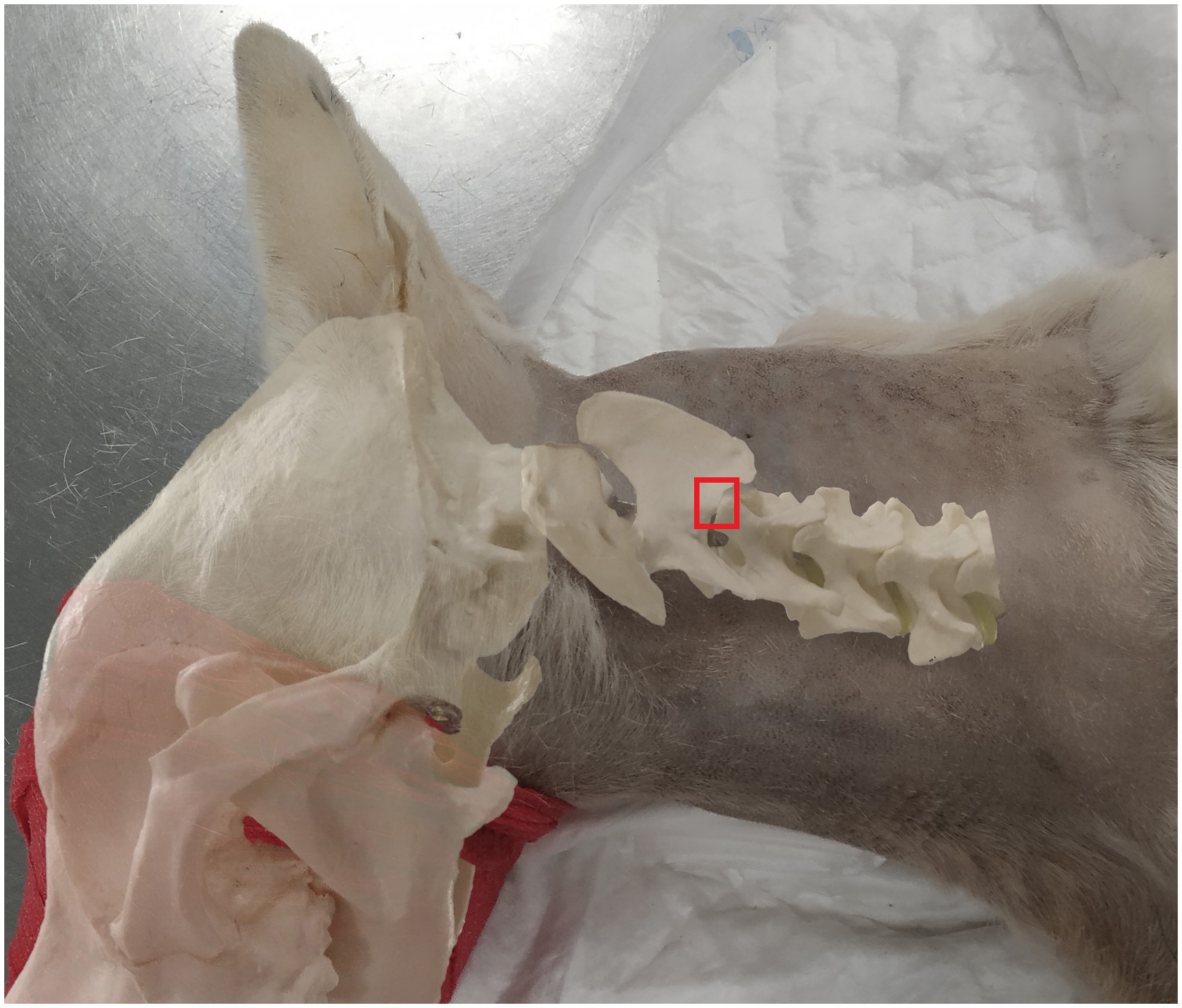
Superimposed bony representation of the cranial cervical region. The anatomical arrangement of the caudal articular process of the axis (red box) to be identified by palpation is shown.

One 37.5 kg female German Shepherd cadaver was included in this phase (Table 1) to correlate the findings from the previous phase and define the guiding US landmarks. After clipping the dorsal and lateral cervical regions, the specimen was positioned in lateral recumbency, with a cushion placed under the neck to ensure vertebral axis alignment. The ultrasound examination was performed by an experienced anesthesiologist (A.C.P.) using a portable linear probe (VINNO Q7L, VINNO Technology, Suzhou, China) connected to a display interface (Surface GO 2, Microsoft, USA); contact gel (Transonic Gel, TELIC, Spain) was applied following skin surface cleaning with alcohol. Prior to scanning, key bony landmarks were identified via palpation: the wing of the atlas, the dorsocaudal border of the spinous process of the axis, and the cervical transverse processes. The caudal articular process of the axis (C2) was selected as the primary external landmark for the injection, which can be palpated approximately at the midpoint between the transverse process and the caudal border of the spinous process of that vertebra (Figure 1; Supplementary Video).

The ultrasound probe was placed cranial to the caudal articular process of the axis, parallel to the longitudinal axis of the vertebral column, with its marker oriented cranially. Angulation and sliding maneuvers were performed to optimize the visualization of the structures of interest. Once identified, the transducer was shifted caudally until the caudal articular process of the axis and its surrounding caudal structures were observed (Supplementary Video). In this position, the optimal needle trajectory for approaching the MCP was simulated.

The first step involved an initial injection performed superficially into the fascial plane between the splenius and semispinalis capitis muscles, termed the sub-splenius plane (SSP), to evaluate whether injectate spread from this superficial plane could consistently reach the MCP and to confirm the ultrasonographic differentiation between these two distinct interfascial planes. A volume of 0.3 mL kg^−1^ of a mixture (C/Cmix; ratio 1:4.5:4.5) consisting of methylene blue (PB.5297, Euromex, Netherlands), iodinated contrast (Ultravist-300, Bayer, Spain), and 0.9% saline solution (FisioVet, B. Braun, Spain) was administered using a 22G 70 mm needle designed for locoregional anesthesia (Lococare, Ecuphar, Spain) attached to a pre-filled syringe. Subsequently, a computed tomography (CT) scan was performed using a Somatom Scope (Siemens, Erlangen, Germany) with a slice thickness of 1–1.5 mm, with the cadaver positioned in sternal recumbency, to evaluate contrast distribution.

**Figure 2:**
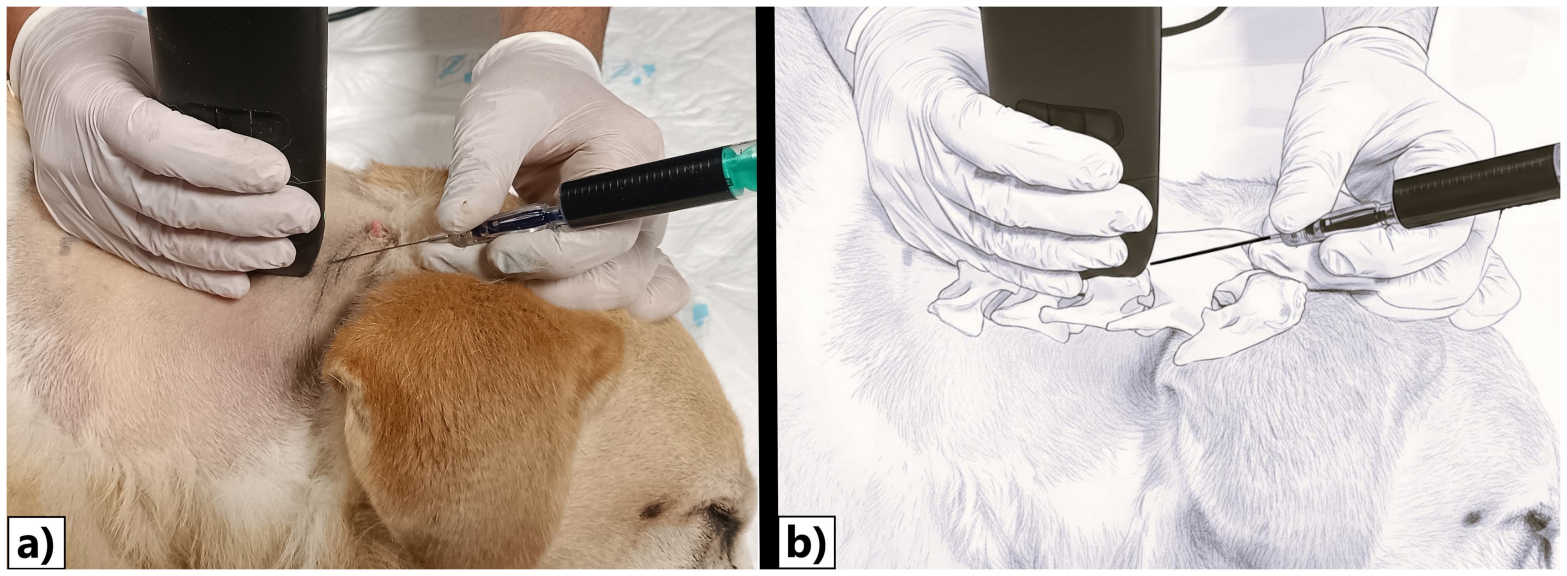
**(a)** Probe positioning and cranio-caudal in-plane approach. **(b)** Schematic superimposition of the cranial cervical vertebrae to show the position over the caudal articular process of the axis.

Thereafter, an US-MCP injection was performed on the contralateral side of the neck in the same cadaver. The probe was placed parallel to the cervical spine, immediately cranial to the caudal articular process of the axis, and shifted caudally until this bony landmark was centered within the ultrasound window. After identifying the semispinalis capitis and multifidus cervicis muscles, as well as the interfascial plane between them, the needle was inserted using an in-plane technique in a cranio-caudal direction (Figure 2). The needle tip was advanced under direct visualization until reaching the interfascial space immediately caudal to the caudal articular process of the axis (Supplementary Video). Once the correct position was confirmed, 0.3 mL kg^−1^ of the C/Cmix was injected.

Immediately after injection, a CT scan was performed, in sternal recumbency and using the equipment previously described. The image evaluation criteria are summarized in Table 2.

**Table 2:** Variables and classification criteria for **(A)** contrast (CT) and **(B)** dye (dissection) distribution.

Variable	Description	Outcome
(A) Computed tomography (CT) evaluation		
1. Target location		
MCP	Presence of contrast in multifidus cervicis plane.	Yes-no
2. Distribution		
Cranial	Cranial limit of vertebral level reached.	
Caudal	Caudal limit of vertebral level reached.	
Dorsal	Spread pattern at the dorsal midline.	No-bilat contact-contralat
Ventral	Spread ventral to the limit of the transverse processes.	Yes-no
3. Spread to Adjacent Structures		
SSP	Degree of spread into the sub-splenius plane.	No-subtle-evident
Lateral foramen	Presence of contrast at the emergence of the C1 nerve.	Yes-no
Epidural space	Presence of contrast within the vertebral canal.	Yes-no
15.6-7.4,-22.3498ptVert. laminae	Contact with vertebral laminae.	Yes-no/affected vertebrae
**(B) Anatomical dissection evaluation**		
1. Nerve staining		
MCP	Stained branches from C2 to C6 in multifidus cervicis plane.	
2. Distribution and extensión		
Dorsoventral	Full plane between transverse and spinous processes or not.	Broad-not broad
Craniocaudal	Extension ≥ C1 to C4 dorsal branch or not.	Broad-not broad
Comm. branches	Presence of communicating structures between branches or not.	Yes-no/indentified branches
3. Spread to adjacent structures		
SSP	Presence of dye into the sub-splenius plane.	Yes-no

Bilat, bilateral; Comm, communicating; Contralat, contralateral; CT, computed tomography; MCP, multifidus cervicis plane; SSP, sub-splenius plane; Vert, vertebral.

Subsequently, a systematic plane-by-plane anatomical dissection was performed on the right side of the neck (US-MCP approach) in left lateral recumbency, followed by the contralateral side (SSP injection). The skin, platysma, cleidocervicalis and rhomboid (capitis and cervicis) muscles were removed to expose the splenius muscle. The latter was detached from the dorsal median raphe and carefully reflected to evaluate the presence of dye within the SSP and to assess whether staining of the lateral branches of the DBCNs occurred. Similarly, the semispinalis capitis muscle (biventer cervicis and complexus) was detached to expose the MCP, which is bounded medially by the multifidus cervicis muscle.

Once exposed, the dye distribution and staining of the DBCNs from C2 to C6 were assessed, considering a nerve staining as complete when the entire circumference was stained over a length of 6 mm, a distance based on the physiological requirements for achieving an effective impulse block (30). Additional evaluation criteria for the distribution and extent of the injectate are detailed in Table 2. Finally, the cadaver was positioned in right lateral recumbency to perform the dissection of the left hemi-neck (SSP injection) following the same protocol, although restricting the evaluation to the detection of dye migration toward the MCP and possible staining of nerve branches at that level.

### 2.3 Phase III: distribution study

Finally, the distribution study was completed following the performance of the standardized US-MCP block in eight cadavers (Table 1).

#### 2.3.1 US-MCP injection

Specimen preparation and positioning were performed strictly following the protocol described in Phase II. In each cadaver, bilateral US-MCP injections were carried out, administering a volume of 0.3 mL kg^−1^ of the C/Cmix. After completing the procedure on one side, the animal was repositioned to perform the contralateral approach. All injections were executed by the same operator (A.C.P.).

#### 2.3.2 CT scan analysis

Immediately upon completion of the bilateral injection in each cadaver, a CT scan was performed. The images were evaluated by a radiologist (N.A.G.) and an anesthesiologist (A.C.P.), applying the criteria defined in Table 2.

#### 2.3.3 Anatomical dissection

Following the CT scan, an anatomical dissection of both sides of the neck was performed. The evaluation during dissection was conducted as described for the multifidus cervicis plane (MCP) in Phase II, applying the criteria defined in Table 2. This assessment was carried out by a clinical anesthesiologist (A.C.P.) and an anatomist (J.T.).

### 2.4 Statistical analysis

All analyses were performed in R (version 4.4.1; R Core Team, 2024, Vienna, Austria). The CT and anatomical dissection datasets were first harmonized at specimen-side level by deriving common binary segment-specific variables indicating whether contrast or dye distribution was present at each cervical level (C2–C6). For CT, segmental involvement was reconstructed from the recorded cranial and caudal limits of spread; for anatomical dissection, segmental involvement was defined directly from the recorded staining status of each cervical dorsal branch. A paired dataset was then created so that, for each cadaver side and cervical segment, CT and anatomical findings could be compared directly within the same specimen.

The primary analysis was descriptive and agreement-based. For each method and cervical segment, the number and percentage of positive specimens were calculated. Segment-by-segment agreement between CT and anatomical dissection was then summarized as the number and percentage of paired observations with concordant findings. For each cervical level, 2 × 2 paired contingency tables were generated to classify observations as both positive, both negative, CT positive/anatomical negative, or CT negative/anatomical positive. These tables were used to quantify concordance and the direction of discordance between methods at each segment.

For visualization, the segment-level dataset was also reshaped into long format, allowing graphical display of the proportion of positive findings by method and segment and heatmap-based representation of specimen-level agreement patterns across cervical levels. Results are presented as counts and percentages. Given the small sample size and the exploratory anatomical nature of the study, the statistical analysis was intentionally focused on paired descriptive comparison and agreement assessment rather than on complex inferential modeling.

## 3 Results

### 3.1 Phase I: anatomical dissection and bibliographic correlation

**Figure 3:**
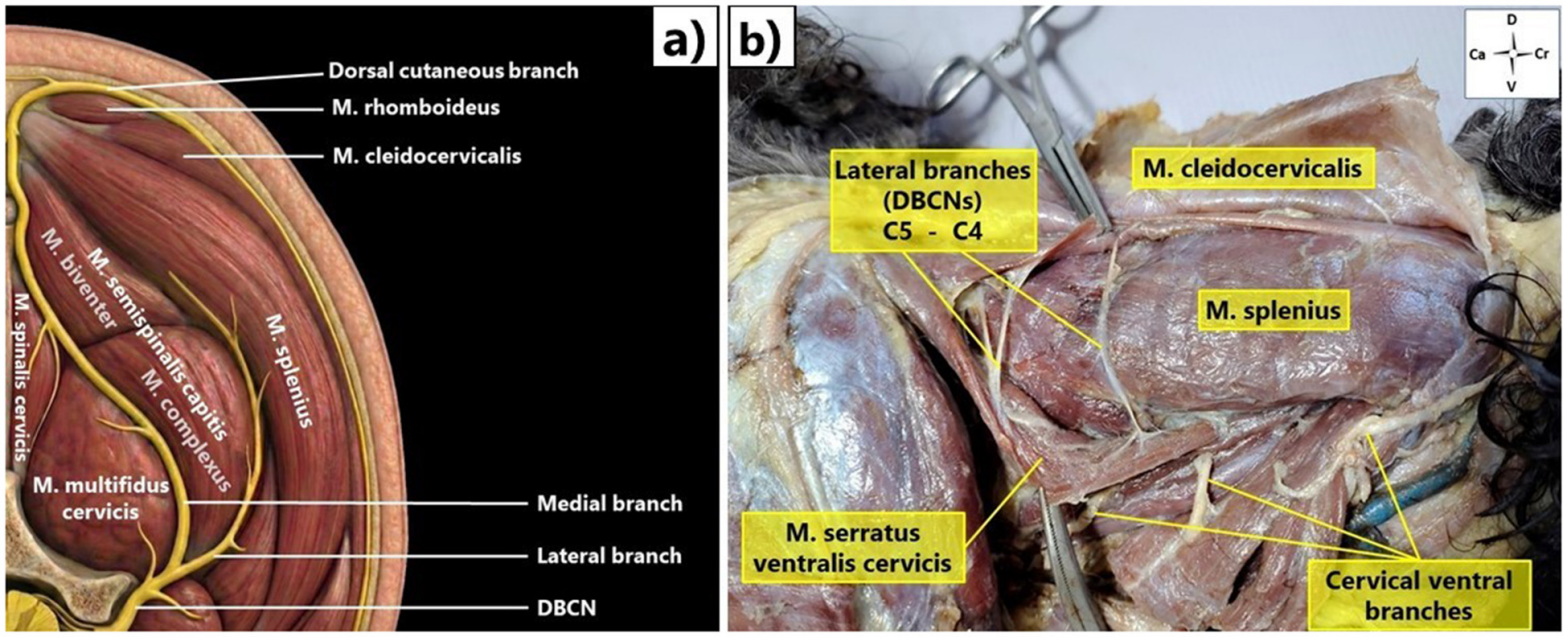
**(a)** Schematic representation of the dorsal branch distribution (at C3 level). The diagram shows the pathway from the dorsal branch to the terminal dorsal cutaneous branch. **(b)** Cervical dissection showing the C4 and C5 lateral branches of the DBCNs (dorsal branches of cervical nerves) on the surface of the splenius m. Ca, caudal; Cr, cranial; D, dorsal; V, ventral.

Specimen dissection allowed for the characterization of the cervical dorsal branch anatomy and the definition of landmarks for the block. Regarding the general nerve conformation, a gradual decrease in the caliber of the DBCNs was observed in a craniocaudal direction. The bifurcation of the dorsal branch into medial and lateral branches was consistently identified (Figure 3a).

The lateral branches, characterized by a smaller diameter, exhibited variable courses depending on the vertebral level; at C2 and C3, they showed a short trajectory, distributing primarily within the splenius muscle. In contrast, the branches at C4 and C5 coursed superficially to the splenius muscle (Figure 3b).

**Figure 4:**
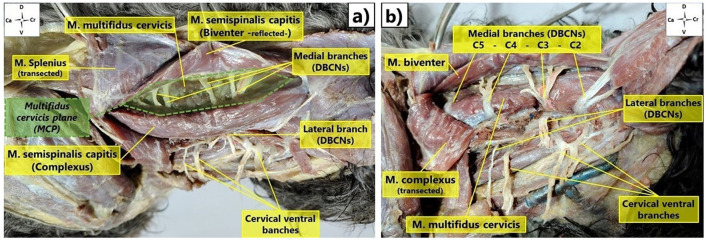
Deep dissection showing the target interfascial plane and medial branches. **(a)** Identification of the multifidus cervicis plane (MCP), located between the semispinalis capitis (complexus) and multifidus cervicis muscles. **(b)** Excision of the complexus m. and lateral reflection of the biventer cervicis m. to fully expose the medial branches of the DBCNs (dorsal branches of cervical nerves) C2–C5. Ca, caudal; Cr, cranial; D, dorsal; V, ventral.

Regarding the medial branches, they were identified as larger-diameter nerves consistently located within the interfascial space bounded medially by the multifidus cervicis muscle and laterally by the semispinalis capititis muscle (complexus and biventer cervicis muscles), as shown in Figure 4.

A consistent finding during dissection was the division of these medial branches into two or three small branches just before penetrating the muscle bellies, continuing their distal course until reaching the skin of the dorsal region as dorsolateral cutaneous branches (Figure 3).

Finally, no macroscopic communicating branches between the nerves were identified in this phase.

### 3.2 Phase II: sonoanatomy and technique standardization

The US study of the dorsolateral neck region allowed for the recognition of the muscular planes and the precise identification of the MCP by its differentiation from the SSP.

**Figure 5:**
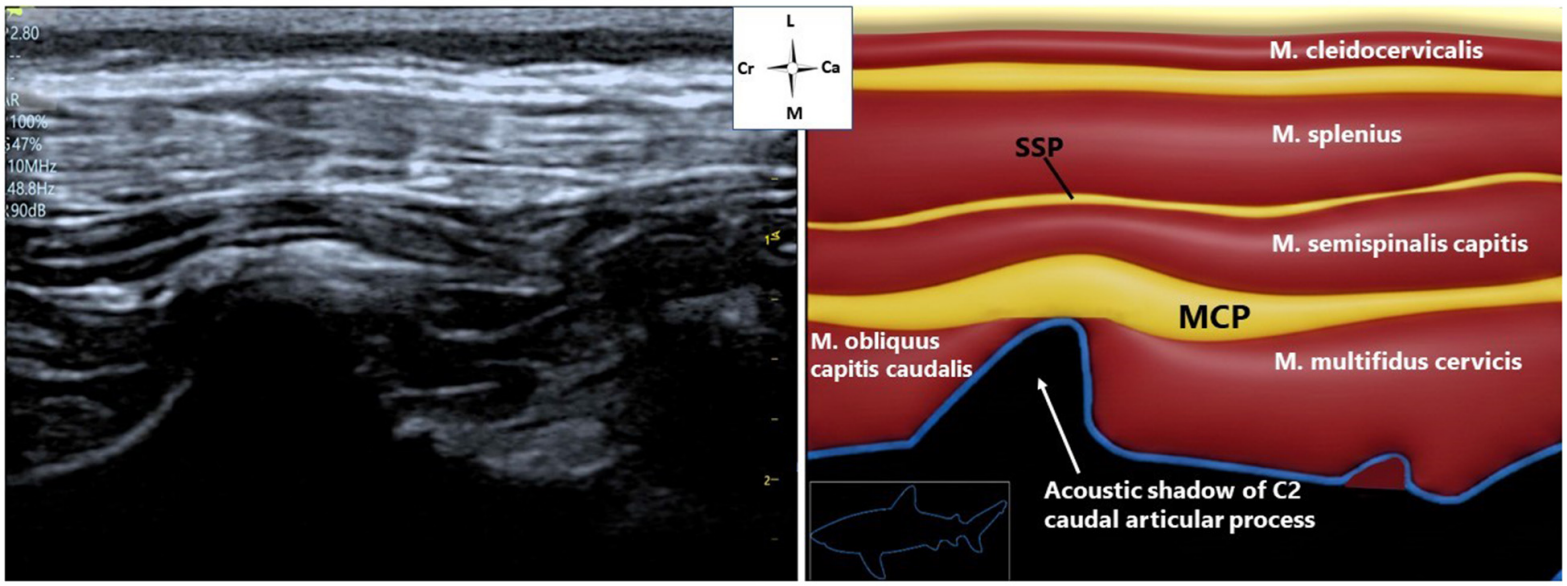
Acoustic window **(left)** and schematic **(right)** showing the target multifidus cervicis plane (MCP), the sub-splenius plane (SSP) and sonoanatomical landmarks. The blue line highlights the shark-shape of the C2 caudal articular process. Ca, caudal; Cr, cranial; L, lateral; M, medial.

Performing a craniocaudal US scanning of the area of interest, the muscle belly of the obliquus capitis caudalis muscle was initially identified. This muscle was observed to taper progressively in a caudal direction, acquiring a wedge-like shape until ending just over the prominence of the caudal articular process of the axis (Figure 5; Supplementary Video).

This process appeared as a triangular structure with a defined hyperechoic border and subsequent acoustic shadowing, creating a silhouette resembling a shark fin; therefore, it was designated as the ‘shark-shape' landmark (Figure 5; Supplementary Video). This structure marked the cranial limit of the scanning area. Immediately caudal to this bony landmark and in a superficial plane, the MCP was identified as an interfascial thickening, bounded medially by the multifidus cervicis and spinalis muscle complex and laterally by the semispinalis capitis muscles. Superficially to the MCP, the SSP was identified, bounded medially by the semispinalis capitis muscle and laterally by the splenius muscle.

#### 3.2.1 SSP injection

During the SSP injection, hydrodissection was observed, with subsequent CT scan confirmation of extensive distribution within this plane. Contrast distribution toward the superficial plane (between the splenius and cleidocervicalis muscles) was identified; however, no contrast migration toward the MCP was observed.

#### 3.2.2 MCP injection

**Figure 6:**
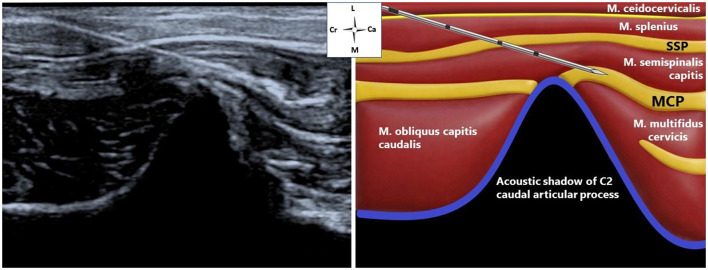
Acoustic window **(left)** and schematic **(right)** showing in-plane approach within the target multifidus cervicis plane (MCP). Ca, caudal; Cr, cranial; L, lateral; M, medial.

Technical validation of the MCP injection in the first cadaver confirmed correct needle visualization using an in-plane approach (Figure 6) and hydrodissection of the space following injection (Supplementary Video). The CT scan showed contrast distribution confined to the target plane, with no distribution ventral to the transverse processes or toward the contralateral dorsal region. Diffusion toward the SSP was observed, but no migration into the spinal canal was detected. Post-imaging anatomical dissection of this cadaver corroborated the staining of the C2 and C3 DBCNs within the MCP, showing extensive dorsoventral distribution. The branches contained within the SSP were also stained.

### 3.3 Phase III: distribution study

Eight cadavers were included in this phase. In one cadaver (CAD05), one side of the neck was excluded due to a recent scar with secondary subcutaneous emphysema, which hindered ultrasonographic visualization. Conversely, the contralateral side showed no alterations in sonoanatomical recognition or injection and was therefore included in the analysis. Consequently, a total of 15 hemi-neck injections were included in this phase.

#### 3.3.1 US-MCP injection

The sonoanatomical landmarks and the MCP were identified in all cases, with a 100% success rate for injections performed within the target plane. The median needle insertion depth to reach the interfascial space was 1.0 (range: 0.8–1.3 cm). Furthermore, the caudal interfascial spread of the injectate was clearly visualized in real-time under US-guidance.

#### 3.3.2 CT scan

**Figure 7:**
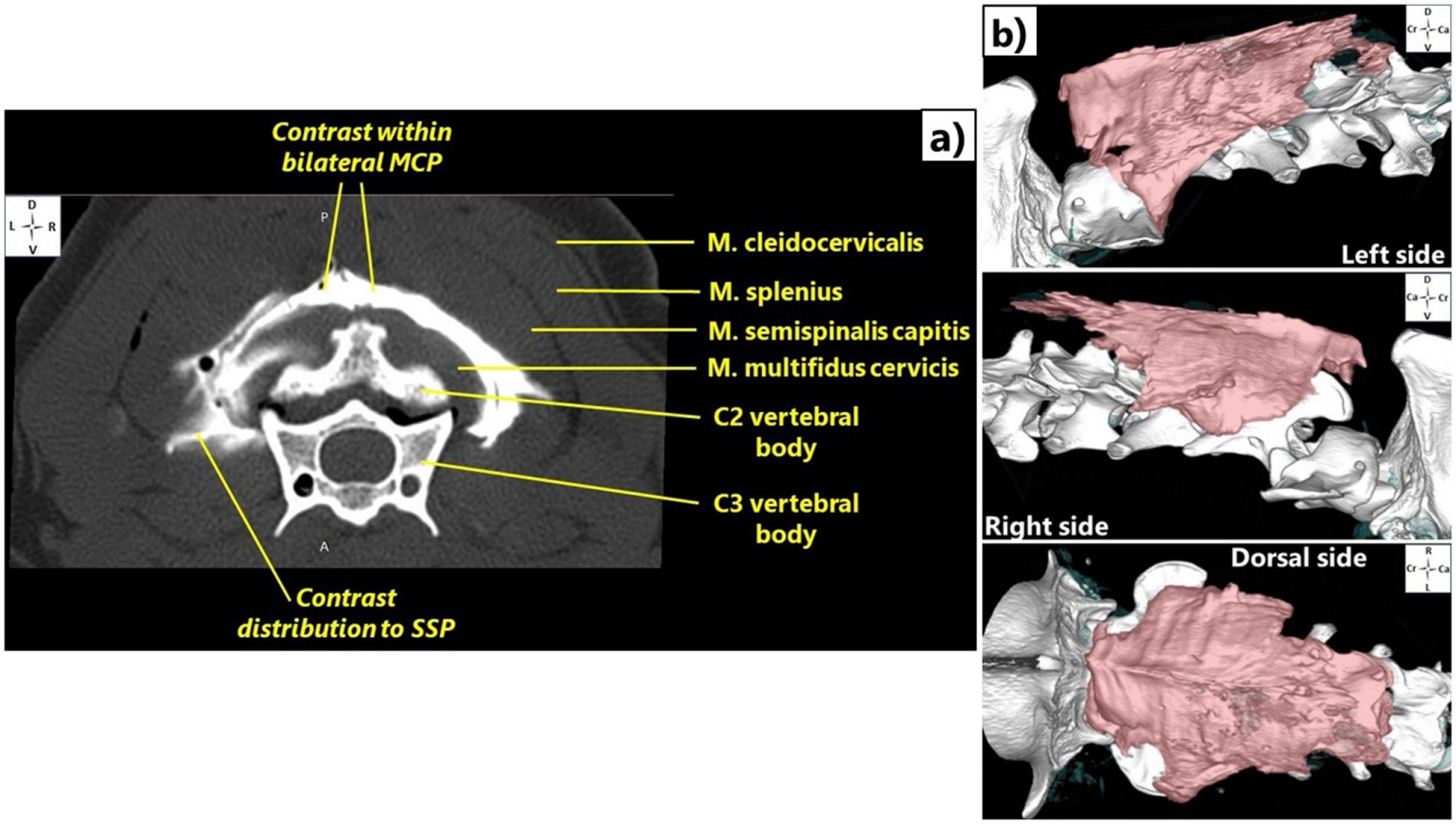
**(a)** Axial CT at C2–C3 showing contrast distribution within the MCP and migration to the SSP. **(b)** 3D CT reconstruction showing bilateral contrast spread (pink) from C1 to C5; views from top to bottom: left side; right side and dorsal view. Ca, caudal; Cr, cranial; D, dorsal; L, left; R, right; V, ventral.

Figure 7 presents a representative CT scan illustrating the contrast distribution pattern. The contrast distribution within the MCP was confirmed in all cases (15/15). Cranially, the injectate reached the C1 or C2 level, while the caudal limit in most cases ranged between C4 and C5. Regarding ventral diffusion, distribution around the obliquus capitis caudalis muscle at the C2 level was observed in 8/15 cases; in 7 of these cases, the contrast partially covered the dorsal lamina of C2. Furthermore, no distribution to ventral interfascial spaces (such as the cervical plexus interfascial plane) or ventral migration at the remaining vertebral levels was observed.

Furthermore, the contrast reached the dorsal midline in most specimens (12/15). It is noteworthy that while bilateral injections showed a visual confluence of contrast at the midline, in the unilateral case, the injectate remained strictly within the medial boundary, with no evidence of contralateral migration. Contrast diffusion into the SSP was observed in all cases, being clearly evident in 9/15 injections.

**Figure 8:**
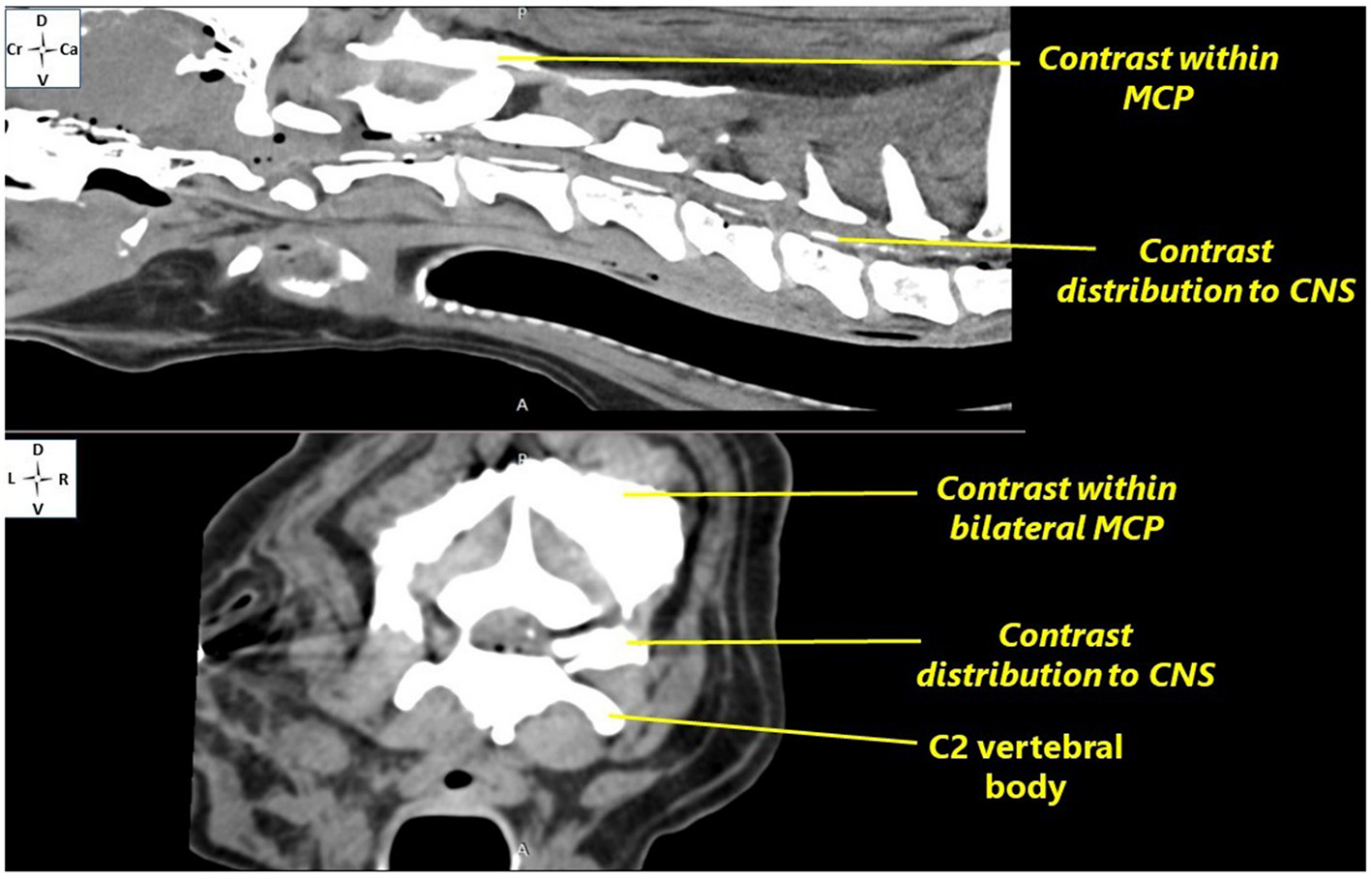
Sagittal **(top)** and axial **(bottom)** CT views showing contrast migration. While contrast is present within the MCP, unintended migration to the central nervous system (CNS) is observed at the C2 level. Ca, caudal; Cr, cranial; D, dorsal; L, left; R, right; V, ventral.

Lastly, no contrast was detected within the lateral vertebral foramen of the atlas in any specimen. However, epidural migration into the spinal canal was observed in 4/15 cases, primarily localized at the C2–C3 intervertebral space; in one of these cases, extensive longitudinal epidural migration was noted from C2 in a caudal direction (Figure 8). Detailed results are presented in Supplementary Table 1.

#### 3.3.3 Anatomical dissection

**Figure 9:**
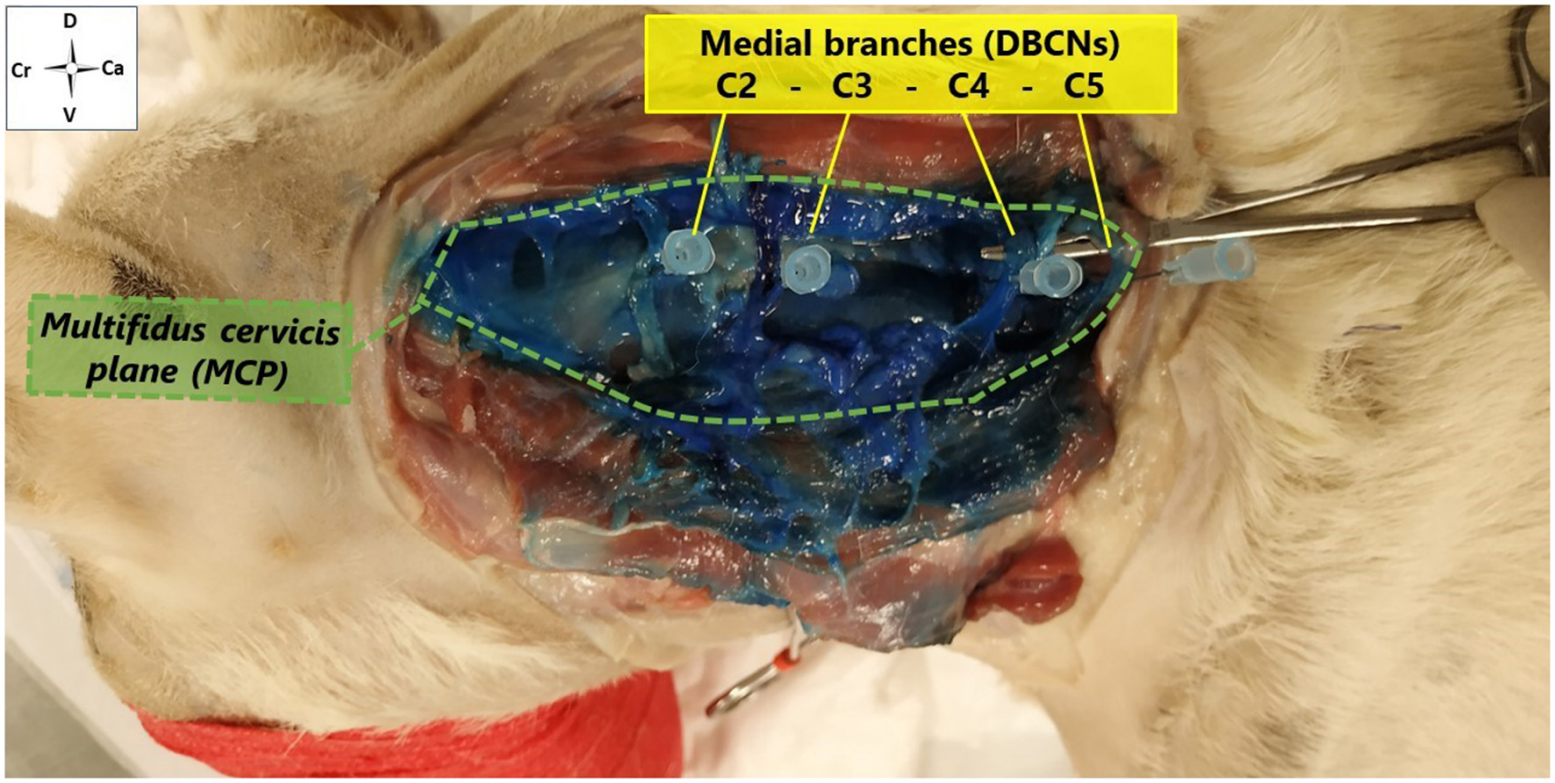
Gross dissection of the cervical region showing methylene blue staining within the multifidus cervicis plane (MCP) and the medial branches (C2–C5). Ca, caudal; Cr, cranial; D, dorsal; DBCNs, dorsal branches of cervical nerves; V, ventral.

Dissection corroborated the interfascial distribution of the dye within the MCP across all specimens (15/15). Extensive dorsoventral spread of the dye was observed in all specimens, while craniocaudal dispersion was extensive in 12/15 cases (Figure 9).

Regarding the evaluation of DBCNs within the MCP, staining of the C2 and C3 was achieved in 100% of the cases (15/15). The C4 was completely stained in 9/15 cases, with one additional case showing partial staining (50% of the nerve circumference). Staining of the C5 was observed in only 2/15 cases (Figure 9), while no staining of the C6 was recorded in any specimen. Regarding the lateral branches within the SSP, staining was observed in 8/15 injections. Finally, communicating branches were identified between C2 and C3 in 7/15 cases, and between C3 and C4 in a single case. Detailed results are presented in Supplementary Table 1.

**Figure 10:**
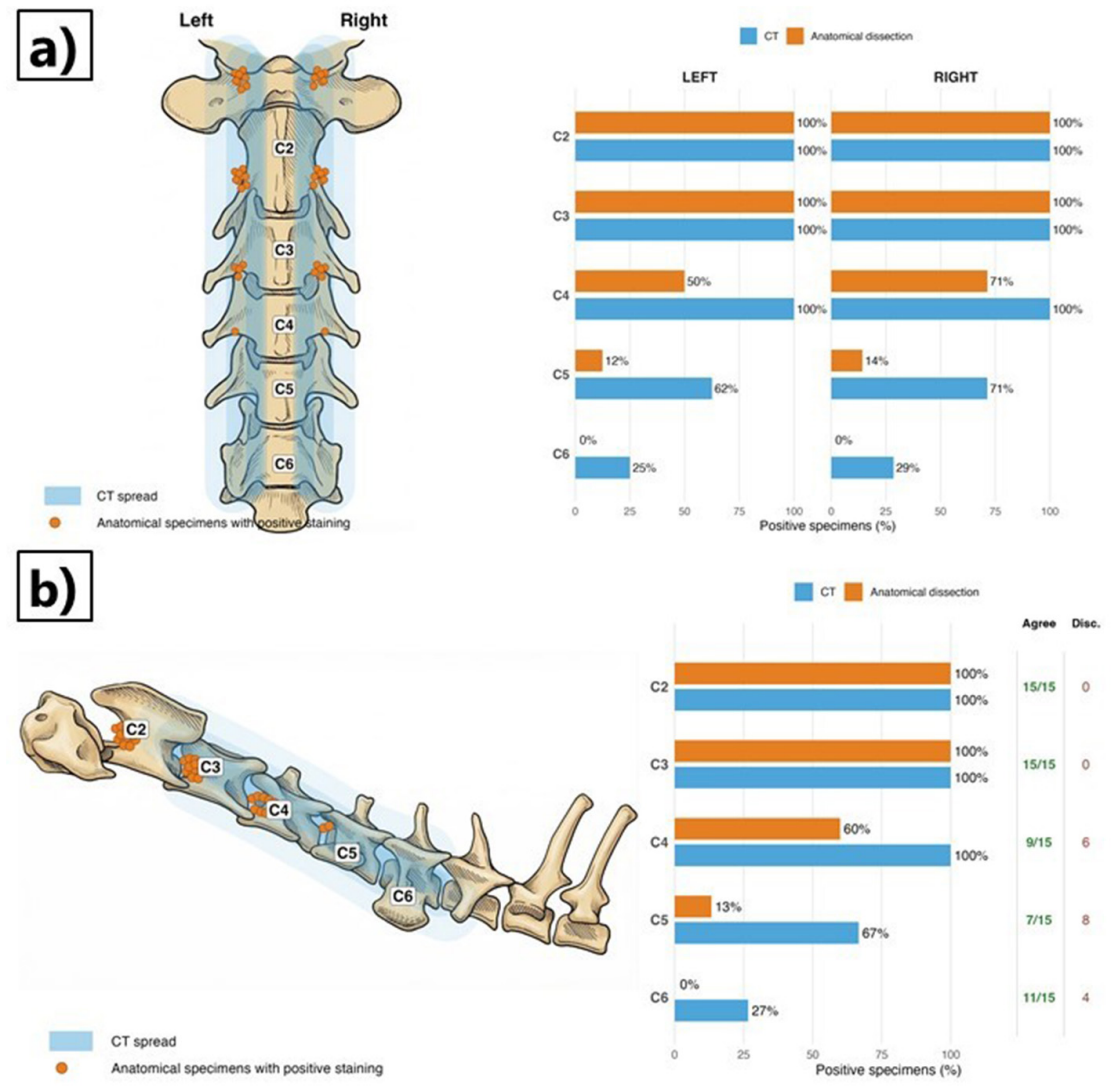
Distribution and success rates of the US-MCP block across cervical levels C2–C6. **(a)** Dorsal anatomical representation of distribution **(left)** and success rates per side **(right)**. **(b)** Lateral anatomical representation of distribution **(left)** and total success with agreement analysis **(right)**. CT, computed tomography; n, number of injections.

Fifteen specimen-sides were available for paired comparison between computed tomography (CT) and anatomical dissection across cervical segments C2–C6. Segment-by-segment analysis showed a clear cranio-caudal gradient, with complete concordance at the more cranial levels and progressively greater divergence at the more caudal levels (Figure 10; Supplementary Table 2).

At C2 and C3, both methods identified spread in all 15/15 specimen-sides (100%), and agreement was complete at both levels, with all paired observations being positive by both CT and anatomical dissection and no discordant findings (Supplementary Table 2).

At C4, CT identified distribution in all 15/15 specimen-sides (100%), whereas anatomical dissection demonstrated positive staining in 9/15 (60%). Accordingly, overall agreement at this level was 9/15 (60%). All concordant observations were double-positive, and the six discordant cases were CT-positive but anatomy-negative; no specimen-side was negative on CT and positive on anatomical dissection (Supplementary Table 2).

Agreement decreased further at C5. CT showed segmental distribution in 10/15 specimen-sides (66.7%), whereas anatomical dissection was positive in only 2/15 (13.3%). Overall agreement was 7/15 (46.7%), comprising two specimen-sides positive by both methods and five negative by both methods. The remaining eight paired observations were discordant, all of them CT-positive and anatomy-negative (Supplementary Table 2).

At C6, CT still demonstrated distribution in 4/15 specimen-sides (26.7%), whereas anatomical dissection did not identify positive staining in any specimen-side (0%). Despite this, overall agreement at C6 was 11/15 (73.3%), driven entirely by 11 concordant double-negative observations. The four discordant pairs were again exclusively CT-positive and anatomy-negative (Supplementary Table 2).

Regarding the comparison of the two methods, a 100% agreement between CT and anatomical dissection was observed at the cranial cervical levels. At the C4–C6 levels, the extent of spread suggested by CT was greater than that confirmed by anatomical staining in all cases. This observed difference was unidirectional throughout the study: no segment identified as negative on CT was found to be positive on anatomical dissection (100% negative predictive value). This pattern is illustrated in the segment-level agreement plots and in the specimen-level heatmap, which show that disagreement became increasingly frequent toward the caudal cervical segments (Figure 10; Supplementary Table 2).

## 4 Discussion

The results obtained in the present study confirm our hypothesis, demonstrating that the US-MCP injection using a cranial approach is a feasible technique that allows for adequate injectate distribution over the cranial DBCNs (C2–C4) in dogs. The consistent sonoanatomical identification of the caudal articular process of the axis, visualized as a triangular hyperechoic bony landmark (“shark-sign”), facilitated precise and reproducible injections. This resulted in a 100% staining rate of the C2 and C3 and 60% for C4, which are key target structures for the innervation of the cranial cervical region.

Regarding the regional anatomy, our observations align with the classical descriptions by Barone (6) concerning the craniocaudal decrease in DBCN caliber and the bifurcation patterns described by Barone and Evans and de Lahunta (6, 7). The consistent localization of the medial branches within the interfascial space between the multifidus cervicis and semispinalis capitis muscles validates these anatomical descriptions (6, 7), confirming this plane as the natural anatomical corridor for the MCP injection. Notably, the superficial course of the lateral branches observed at C4 and C5 represents a variation not previously reflected in the consulted literature. Furthermore, the absence of macroscopic communicating branches in Phase I contrasts with the descriptions of a “dorsal cervical plexus” by König (31) and Singh (32), a point that is further analyzed later in this discussion considering the findings of Phase III.

Cervical pain is a common clinical presentation in small animal practice. According to Trevail and Behr (3), it is commonly associated with conditions affecting the C1–C5 segments. This condition is particularly relevant in cases such as intervertebral disc disease, where associated pain is generally classified as moderate to severe (33, 34). Although systemic opioids and non-steroidal anti-inflammatory drugs remain the cornerstone of analgesia management, their use entails dose-dependent adverse effects such as dysphoria, bradycardia, and renal/gastrointestinal disturbances (35, 36). These limitations underscore the need for multimodal analgesia strategies, where locoregional and interventional techniques are essential to optimize perioperative control in spinal surgery (37) and improve the management of complex spinal pain (38). In this scenario, as widely described in human medicine, fascial plane blocks have emerged as an alternative with a potentially superior safety profile by depositing the anesthetic in avascular compartments relatively distant from the neuraxis (39). This safety profile facilitates their inclusion in Enhanced Recovery After Surgery (ERAS) protocols, a clinical trend already being successfully adopted in veterinary medicine (10). Nevertheless, the potential for epidural migration observed in the present study suggests that this safety profile should be evaluated with caution at least in this approach, as the assumption of total isolation from the neuraxis may not strictly hold in the cervical region.

Recently, Herrera-Linares et al. (22) described the inter-transversospinalis plane (ITP) block which, despite the different nomenclature, targets an anatomically comparable plane to the MCP. However, their technique performed a more caudal approach (C5) using two different volumes; their high volume is equivalent to the one used in the present study. Their results revealed extensive craniocaudal spread with broad staining of the DBCNs; nevertheless, C2 was not stained in all cases, even with higher volumes. In contrast, our approach centered directly at the C2 level resulted in 100% staining of the C2 and C3 segments, with relevant 60% staining at C4, while only reaching 13.33% at C5. This difference in distribution could be attributed not only to the distinct injection site but also to the complex fascial organization of the region. Recent human medicine studies on the deep cervical plane (Cervical Cervicis Plane, CCeP) block have hypothesized the existence of an ‘incomplete functional barrier' at the C2 level (40). If an analogous structure exists in dogs within the MCP, it could limit the cranial spread of injectate, thereby justifying the use of a cranial approach to ensure successful blockade of the high cervical segments.

When comparing the US-MCP approach with other regional techniques described in dogs, several anatomical and clinical distinctions emerge depending on the surgical target. While the cervical plexus block could be indicated for ventrolateral procedures (25), it would not be expected to provide adequate coverage for the dorsal midline. Regarding the erector spinae plane (ESP) block, its application in the canine cervical region remains largely unexplored (27); theoretically, it could provide broad coverage of both medial and lateral branches through a deeper approach reaching the bony landmarks, whereas the MCP targets a more superficial interfascial plane. Furthermore, regarding the SSP approach, although its distribution in this region has yet to be investigated, the MCP injection targets the medial branches of the DBCNs; since these are the most developed and provide cutaneous ramifications, the MCP approach could potentially allow a more comprehensive distribution than an injection in the SSP, which would likely only affect the lateral branches if no MCP distribution is obtained.

In terms of procedural feasibility, the US-MCP approach is a highly reproducible technique with a relatively straightforward learning curve for operators familiar with ultrasound-guided interfascial injections. The consistent visualization of the ‘shark-sign' provides a reliable bony landmark that simplifies the procedure and ensures the correct level of injection, resulting in a moderate level of technical difficulty. Nevertheless, the proximity to the neuraxis requires precise needle handling to ensure the tip remains within the interfascial space, thereby avoiding accidental puncture of deeper anatomical structures.

Regarding epidural migration, both studies highlight the anatomical complexity of the cranial cervical region and the potential permeability toward the neuraxis. While Herrera-Linares et al. (22) reported contrast migration into the vertebral canal in two cases, epidural migration was detected in 4 out of 15 specimens in our study. Although this was mostly characterized as focal and circumscribed, one specimen exhibited extensive multisegmental migration originating at the C2–C3 level. This observation supports the hypothesis of lower tissue resistance proposed by Herrera-Linares, suggesting that this level constitutes an intrinsic anatomical vulnerability in dogs, regardless of the technique, a phenomenon not yet reported in human medicine for this specific approach. Clinical consequences of unintended epidural distribution in the cranial cervical region may include, for example, Horner's syndrome, hypotension due to sympathetic blockade, cardiovascular and respiratory depression, or affectation of the central nervous system (41–43).

From an anatomical perspective, the identification of communicating branches between the DBCNs of contiguous segments in several specimens during Phase III warrants further discussion. This finding, although inconsistently observed, aligns with classical veterinary literature regarding the existence of a ‘dorsal cervical plexus' (31, 32), illustrating the multisegmental contribution of dorsal branches to the innervation of this region. However, it should be noted that macroscopic observation only confirms the presence of these communicating branches. To confirm axonal intercommunication and rule out a simple shared epineural pathway, specialized techniques such as fascicular microdissection, electrophysiological conduction studies, or axonal tracing would be required.

From a clinical perspective, the consistent coverage of the C2 and C3 dorsal branches, along with the considerable coverage of C4, suggests high potential for regional anesthesia in cranial dorsal surgical approaches. This could be useful for both spinal procedures, such as hemilaminectomies for disc extrusions, common in small breeds (44) or dorsal atlantoaxial stabilization (45), as well as dorsolateral soft tissue interventions like biopsies, tumor excisions, or post-traumatic reconstructions. Indeed, this block has already been reported as part of a multimodal approach in a C3 dorsal laminectomy case, where adequate analgesic management and hemodynamic stability were observed without associated complications (29). Beyond surgery, and extrapolating its use from human medicine for myofascial pain (21) or cervicogenic headache (46), the technique could open new therapeutic options for managing refractory chronic pain in dogs.

Finally, the inherent limitations of the cadaveric model must be acknowledged. As noted in the literature, post-mortem alterations in tissue tension and biomechanical properties of fascial planes differ from *in vivo* conditions, potentially influencing fluid diffusion dynamics (47). Furthermore, the behavior of the dye and methylene blue mixture may differ from the diffusion of local anesthetics. Recent evidence suggests that lipophilic drugs, such as bupivacaine, possess a significantly higher permeation capacity across fascial barriers compared to visual markers, which tend to remain restricted to the injection compartment (48). Moreover, the 6 mm staining threshold was adopted based on the physiological requirement to expose at least three consecutive nodes of Ranvier to reliably interrupt saltatory conduction (30). Although Raymond et al. suggest that exposure beyond this length is not a primary determinant of blocking potency, anatomical spread in a cadaveric model remains a proxy and does not guarantee functional blockade. Consequently, its clinical efficacy remains to be validated in live animals. Consequently, its clinical efficacy remains to be validated in live animals. Additionally, although potential epidural spread was assessed via CT imaging, it was not further assessed during dissection as the spinal canal was not accessed. The lack of cryosectioning techniques at the time of the study also limited a more detailed anatomical visualization of the injectate's most medial distribution. Lastly, while the selected volume corresponds to those typically used in canine interfascial block studies, further research is required to define the optimal volume. Future studies evaluating lower injection volumes would be of particular interest, as they might reduce the likelihood of contralateral spread and mitigate the risk of unintended epidural migration, thereby assessing the block's efficacy and safety in clinical settings.

## 5 Conclusion

In conclusion, the described US-guided MCP approach constitutes a feasible and highly specific technique for the desensitization of the cranial dorsal cervical region. The consistent staining observed in the C2 and C3 dorsal branches, and potentially C4, supports its potential as a promising tool for integration into multimodal analgesia protocols for this region.

## Data Availability

The original contributions presented in the study are included in the article/Supplementary material, further inquiries can be directed to the corresponding author.
