# Supplementary material for: Ultrasound-guided cranial multifidus cervicis plane block in dogs: a cadaveric study
Source: Front Vet Sci. 2026 May 26;13:1835968. doi: 10.3389/fvets.2026.1835968 (PMC13246387; doi:10.3389/fvets.2026.1835968)
Supplement: Supplementary file 1 [file Table_1.docx]

Supplementary Material

# Supplementary Tables

**Supplemmentary Table 1.** Summary of anatomical dissection and contrast distribution results according to Table 2 criteria (n = 15). C. Branc, communicating branch; CrCa, craniocaudal; DBCNs, dorsal branches of cervical nerves; DV, dorsoventral; L, left; MCP, multifidus cervicis plane; R, right; SSP, superficial splenius plane.

| **CT-results** | | | | | | | | | |
| --- | --- | --- | --- | --- | --- | --- | --- | --- | --- |
| **ID** | **MCP** | **Contrast Distribution** | | | | **Spread to Adjacent Structures** | | | |
|  |  | **Cranial** | **Caudal** | **Dorsal** | **Ventral** | **SSP** | **Lateral foramen** | **Vertebral  Laminae** | **Epidural Space** |
| **CAD02 L** | YES | C2 | C6 | SI | NO | YES. Subtle | NO | NO | NO |
| **CAD02 R** | YES | C2 | C5 | SI | NO | YES. Evident | NO | NO | NO |
| **CAD03 L** | YES | C2 | C5 | SI | YES * | YES. Evident | NO | YES. C2 | NO |
| **CAD03 R** | YES | C2 | C5 | SI | NO | YES. Evident | NO | NO | NO |
| **CAD04 L** | YES | C1 | C6 | SI | NO | YES. Subtle | NO | NO | NO |
| **CAD04 R** | YES | C2 | C5 | SI | NO | YES. Subtle | NO | NO | YES. C2-C3 |
| **CAD05 L** | YES | C1 | C5 | NO | YES * | YES. Evident | NO | YES. C2 | NO |
| **CAD06 L** | YES | C1 | C4 | SI | YES * | YES. Evident | NO | NO | NO |
| **CAD06 R** | YES | C1 | C6 | SI | NO | YES. Subtle | NO | NO | NO |
| **CAD07 L** | YES | C1 | C5 | SI | YES * | YES. Evident | NO | YES. C2 | YES. C2-C3 |
| **CAD07 R** | YES | C2 | C7 | SI | NO | YES. Subtle | NO | NO | NO |
| **CAD09 L** | YES | C2 | C4 | SI | YES * | YES. Evident | NO | YES. C2 | YES. C2-C3 |
| **CAD09 R** | YES | C2 | C4 | SI | YES * | YES. Evident | NO | YES. C2 | NO |
| **CAD10 L** | YES | C1 | C4 | NO | YES * | YES. Subtle | NO | YES. C2 | NO |
| **CAD10 R** | YES | C1 | C4 | NO | YES * | YES. Subtle | NO | YES. C2-C3 | YES. C2-C3/C3-C4 |
| * Sorrounding Oblicuus capitis caudalis m. | | | | | | | | | |
|  |  |  |  |  |  |  |  |  |  |
| **Anatomical dissections results** | | | | | | | | | |
| **ID** | **MCP** | **Dye Distribution and C.Branch** | | | **DBCNs staining in MCP** | | | | |
|  |  | **DV** | **CrCa** | **C.Branch** | **C2** | **C3** | **C4** | **C5** | **C6** |
| **CAD02 L** | YES | BROAD | BROAD | NO | YES | YES | YES | YES | NO |
| **CAD02 R** | YES | BROAD | BROAD | YES. C2-C3 | YES | YES | YES | NO | NO |
| **CAD03 L** | YES | BROAD | BROAD | NO | YES | YES | YES | NO | NO |
| **CAD03 R** | YES | BROAD | BROAD | NO | YES | YES | YES | NO | NO |
| **CAD04 L** | YES | BROAD | BROAD | NO | YES | YES | YES | NO | NO |
| **CAD04 R** | YES | BROAD | BROAD | YES. C3-C4 | YES | YES | NO | NO | NO |
| **CAD05 L** | YES | BROAD | BROAD | NO | YES | YES | NO | NO | NO |
| **CAD06 L** | YES | BROAD | NOT BROAD | YES. C2-C3 | YES | YES | NO | NO | NO |
| **CAD06 R** | YES | BROAD | BROAD | YES. C2-C3 | YES | YES | NO | NO | NO |
| **CAD07 L** | YES | BROAD | NOT BROAD | NO | YES | YES | NO | NO | NO |
| **CAD07 R** | YES | BROAD | BROAD | NO | YES | YES | YES | YES | NO |
| **CAD09 L** | YES | BROAD | NOT BROAD | YES. C2-C3 | YES | YES | NO | NO | NO |
| **CAD09 R** | YES | BROAD | BROAD | YES. C2-C3 | YES | YES | YES | NO | NO |
| **CAD10 L** | YES | BROAD | BROAD | YES. C2-C3 | YES | YES | YES | NO | NO |
| **CAD10 R** | YES | BROAD | BROAD | YES. C2-C3 | YES | YES | YES | NO | NO |

**Supplementary Table 2.** Segment-by-segment agreement and discordance between computed tomography (CT) and anatomical dissection across cervical levels C2-C6. CT, computed tomography; n, number of injections.

| **Segment** | | **C2** | **C3** | **C4** | **C5** | **C6** |
| --- | --- | --- | --- | --- | --- | --- |
| **n** | | 15 | 15 | 15 | 15 | 15 |
| **CT, n (%)** | | 15 (100.0) | 15 (100.0) | 15 (100.0) | 10 (66.7) | 4 (26.7) |
| **Anatomical dissection, n (%)** | | 15 (100.0) | 15 (100.0) | 9 (60.0) | 2 (13.3) | 0 (0.0) |
| **Paired  findings** | **Agreement, n (%)** | 15 (100.0) | 15 (100.0) | 9 (60.0) | 7 (46.7) | 11 (73.3) |
|  | **Both positive, n** | 15 | 15 | 9 | 2 | 0 |
|  | **Both negative, n** | 0 | 0 | 0 | 5 | 11 |
|  | **CT positive / Anatomical dissection negative, n** | 0 | 0 | 6 | 8 | 4 |
|  | **CT negative / Anatomical dissection positive, n** | 0 | 0 | 0 | 0 | 0 |

**Supplementary Video 1.** Anatomical landmarks, dissection, and utrasound-guided approach of the Multifidus Cervicis Plane (MCP).
